# A Copper (II) Ensemble-Based Fluorescence Chemosensor and Its Application in the ‘Naked–Eye’ Detection of Biothiols in Human Urine

**DOI:** 10.3390/s20051331

**Published:** 2020-02-29

**Authors:** Yue Wang, Huan Feng, Haibo Li, Xinyi Yang, Hongmin Jia, Wenjun Kang, Qingtao Meng, Zhiqiang Zhang, Run Zhang

**Affiliations:** 1 School of Chemical Engineering, University of Science and Technology Liaoning, Anshan 114051, China; Wangyue9088@163.com (Y.W.); yxz0601y@163.com (X.Y.); jhongmin66@163.com (H.J.); 2 Shandong Provincial Key Laboratory of Chemical Energy Storage and Novel Cell Technology, Department of Chemistry, Liaocheng University, Liaocheng 252059, China; haiboli@mail.ustc.edu.cn (H.L.); kangwenjun@lcu.edu.cn (W.K.); 3 Australian Institute for Bioengineering and Nanotechnology, The University of Queensland, Brisbane 4072, Australia; r.zhang@uq.edu.au

**Keywords:** chemosensor, biothiols, displacement, detection, urine samples

## Abstract

Quick and effective detection of biothiols in biological fluids has gained increasing attention due to its vital biological functions. In this paper, a novel reversible fluorescence chemosensor (**L**-Cu^2+^) based on a benzocoumarin-Cu^2+^ ensemble has been developed for the detection of biothiols (Cys, Hcy and GSH) in human urine. The chemosensing ensemble (**L**-Cu^2+^) contains a 2:1 stoichiometry structure between fluorescent ligand **L** and paramagnetic Cu^2+^. **L** was found to exclusively bond with Cu^2+^ ions accompanied with a dramatic fluorescence quenching maximum at 443 nm and an increase of an absorbance band centered at 378 nm. Then, the in situ generated fluorescence sluggish ensemble, **L**-Cu^2+^, was successfully used as a chemosensor for the detection of biothiols with a fluorescence “OFF-ON” response modality. Upon the addition of biothiols, the decomplexation of **L**-Cu^2+^ led to the liberation of the fluorescent ligand, **L**, resulting in the recovery of fluorescence and absorbance spectra. Studies revealed that **L**-Cu^2+^ possesses simple synthesis, excellent stability, high sensitivity, reliability at a broad pH range and desired renewability (at least 5 times). The practical application of **L**-Cu^2+^ was then demonstrated by the detection of biothiols in human urine sample.

## 1. Introduction

Biothiols, such as cysteine (Cys), homocysteine (Hcy) and glutathione (GSH), play critical roles in cellular processes and are always involved in physiological and pathological actions [1,2,3]. It is widely reported that cysteine (Cys) and homocysteine (Hcy) are associated with cellular growth, while glutathione (GSH) is mainly related to cellular homeostasis [4]. Abnormal levels of cellular biothiols are implicated in a variety of diseases, such as leucocyte loss, psoriasis, liver damage, slowed growth, asthma, cancer and acquired immune deficiency syndrome (AIDS) [5]. For example, the deficiency of Cys may lead to symptoms like hair depigmentation, muscle loss and slow growth [6,7,8]. Elevated level of Hcy in human plasma can increase the risk of diseases like osteoporosis, neural tube defects and even Alzheimer’s disease [9,10]. GSH, as the most abundant cellular biothiol, might cause oxidative stress and some other serious illness including cancers and AIDS [11,12,13]. In view of their importance, developing safe, highly specific and sensitive detection methods for biothiols in living systems is strongly desired in biochemistry and biomedicine research fields [14].

Among various conventional methods such as electrochemical approaches [15,16], high-performance liquid chromatography (HPLC) [17,18], gas chromatography [19,20] and mass spectrometry (MS) [21], fluorescence analysis using a responsive molecular probe or chemosensor has been recognized as one of the most promising approaches due to its excellent sensitivity, selectivity, and capability of detecting analytes in live biological specimens [22,23,24,25,26]. To date, a large number of fluorescence chemosensors aiming to distinguish biothiols from other amino acids and bioactive species have been developed based on several sensing mechanisms: (1) Michael addition [27,28,29]; (2) cyclization with aldehyde [30,31]; (3) the cleavage of sulfonamide, sulfonate esters, selenium-nitrogen bonds and disulfide bonds [32,33,34,35,36,37,38]; (4) intramolecular elimination [39,40]. However, most organic reaction-based fluorescence chemodosimeters suffer several problems such as time-consuming synthesis and the synthesis procedure commonly requires strict reaction condition, which somehow limit the practical application in biosystems [41]. Moreover, to the best of our knowledge, few of them could achieve analyzing biothiols in body fluids, such as human urine [42,43].

**Scheme 1:**
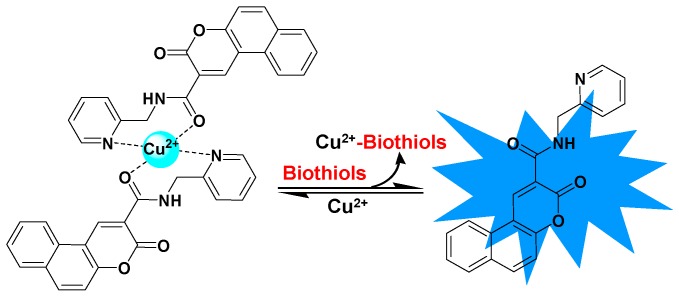
The proposed sensing mechanism of **L**-Cu^2+^ towards biothiols (cysteine (Cys), homocysteine (Hcy) and glutathione (GSH)).

Recently, indicator displacement-based fluorescence chemosensor, as a new strategy, has emerged for the detection of certain biomolecules in living systems. Among them, a Cu^2+^-ensemble based fluorescence chemosensor features simple synthetic route, improved water solubility, a fluorescence “OFF-ON” response pattern as well as desired reversibility [44,45,46]. By the way, our group has been involved in longstanding exploration of the synthesis and application of this class of fluorescence chemosensors [47,48,49,50,51,52,53,54,55,56,57,58,59]. Herein, we designed and synthesized a novel benzocoumarin-Cu^2+^ ensemble based fluorescence chemosensor for the selective detection of biothiols under simulated physiological conditions and in human urine. As described in Scheme 1, the fluorescent ligand (**L**), as expected, could coordinate with Cu^2+^ to form **L**-Cu^2+^ which exhibited nearly no fluorescence owing to the paramagnetic quenching effect of Cu^2+^ [50]. The subsequent ensemble **L**-Cu^2+^ would then be the platform for the real-time detection of biothiols with “OFF–ON”response due to higher affinity between biothiols and Cu^2+^. Additionally, the application of **L**-Cu^2+^ in the detection of biothiols in human urine was successfully demonstrated.

## 2. Materials and Methods

### 2.1. Materials and Instruments

Diethyl malonate, 2-hydroxy-1-naphthaldehyde, pyridin-2-ylmethanamine, 1-(3-dimethylaminopropyl)-3-ethylcarbodimide hydrochloride (EDCI) and 4-(dimethylamino) pyridine (DMAP) were purchased from Aladdin reagent Co. (Shanghai, China). Metal ions (nitrate salts), biothiols and amino acids were obtained from Sinopharm Chemical Reagent Co., Ltd. (China). Slow qualitative filter paper (Φ 9 cm) was purchased from commercial source. Unless otherwise stated, solvents and reagents were of analytical grade from commercial suppliers and were used without further purification. Deionized water was used throughout. 

^1^H nuclear magnetic resonance (NMR) and ^13^C NMR spectra were recorded with an AVANCE600MHZ spectrometer (BRUKER) with chemical shifts reported as *ppm* (in CDCl_3_, TMS as internal standard). Coupling constants (*J* values) are reported in hertz. API mass spectra were recorded on an Agilent 6530 QTOF spectrometer. Absorption spectra were recorded with a Perkin Elmer Lambda 900 ultraviolet/visible/near-infrared (UV/VIS/NIR) spectrophotometer (USA). Fluorescence spectra were measured with Perkin Elmer LS55 luminescence spectrometer (USA). All pH measurements were made with an OHAUS Starter 3100/f meter (USA). 

### 2.2. Synthesis of Fluorescent Ligand L

**L** was gained through a typical amidation reaction [51,52,53,54]. To a solution of benzocoumarin-3-carboxylic acid [55,56,57] (0.239 g, 1 mmol) in 20 mL CH_2_Cl_2_, pyridin-2-ylmethanamine (0.108 g, 1 mmol), 1-(3-dimethylaminopropyl)-3-ethylcarbodiimide hydrochloride (EDCI) (0.383 g, 1 mmol) and a catalytic amount of 4-(dimethylamino)pyridine (DMAP) (12 mg) were added sequentially and the mixture was stirred for 8 h. The solution was washed with water (2 × 8 mL) and dried over anhydrous sodium sulfate, and the solvent was removed under a pressure-reducing condition. The crude product was further purified by column chromatography to obtain **L** in 89% yield. ^1^H-NMR (600 MHz, CDCl_3_) *ó*(*ppm*): 9.67 (s, 2H), 8.63 (d, *J* = 6.72 Hz, 1H), 8.42 (d, *J* = 12.6 Hz, 1H), 8.10 (d, *J* = 13.56 Hz, 1H), 7.93 (d, *J* = 12.12 Hz, 1H), 7.74 (t, *J* = 11.49 Hz, 1H), 7.68 (t, *J* = 11.46 Hz, 1H), 7.61 (t, *J* = 11.22 Hz, 1H), 7.50 (d, *J* = 13.5 Hz, 1H), 7.37 (d, *J* = 11.7 Hz, 1H), 7.21 (t, *J* = 9.18 Hz, 1H), 4.86 (d, *J* = 8.16 Hz, 2H). ^13^C NMR (150 MHz, CDCl_3_): 162.1, 161.3, 156.9, 154.9, 149.5, 143.8, 136.7, 135.8, 130.3, 129.5, 129.1, 126.7, 122.3, 121.9, 121.7, 116.9, 116.3, 113.2, 45.5. High-resolution mass spectrometry (HRMS)-API (positive mode, m/z) for [**L**+H]^+^: calcd 331.1077, found: 331.1074. Mp: 184.4-185.2 °C.

### 2.3. Preparation of the Stock Solutions of L

**L** (16.5 mg, 0.05 mmol) was dissolved in 100 mL of N,N-dimethylformamide (DMF) to prepare a high concentration of the stock solution (0.5 mM). Before spectroscopic measurements, the solution of **L** (10 μM, DMF/HEPES, 7:3, *v/v*, pH = 7.4) was freshly prepared by diluting the high concentration stock solution of **L** using HEPES buffer (20 mM, pH = 7.4). The solutions of various testing biothiols (20 mM) and amino acid (20 mM) were prepared in distilled water.

### 2.4. Quantum Yield Measurement

The fluorescence quantum yields have been measured referring to a reported method [58].

### 2.5. The Study of Reversibility of L-Cu^2+^

The reversibility of **L**-Cu^2+^ was investigated by repeating the processes of binding and release [59]. The experimental process by sequentially adding Cu^2+^ and biothiols to the same solutions was repeated five times, and the volume ratios of Cu^2+^ and biothiols were: Cu^2+^/Cys. 0:0, 10:0, 10:10, 20:10, 20:20, 30:20; 30:30, 40:30, 40:40, 50:40; Cu^2+^/Hcy. 0:0, 10:0, 10:10, 20:10, 20:20, 30:20; 30:30, 40:30, 40:40, 50:40; Cu^2+^/GSH. 0:0, 10:0, 10:5, 20:5, 20:10, 30:10; 30:20, 40:20, 40:30, 50:30, respectively.

### 2.6. Visualization of Biothiols in Human Urine

To 50 mL acetonitrile solution of **L** (0.5 mM, 100 mL), filter papers (25 mm × 75 mm) were immersed for 30 min, followed by dipping into Cu^2+^ solution (0.2 mM, 100 mL) for another 30 min. Then, **L**-Cu^2+^-coated test papers were dried using the blower.

Human urine samples were collected from a healthy volunteer and informed consent was obtained from the volunteer prior to sample collection. All experiments were performed in compliance with the relevant laws and institutional guidelines. For “naked-eye” analysis of biothiols in human urine samples, **L**-Cu^2+^-coated test paper was immersed in urine samples and was photographed under 365 nm UV light.

## 3. Results

### 3.1. Design, Synthesis of Fluorescent Ligand (L)

The fluorescent ligand (**L**) was designed by introducing pyridin-2-ylmethanamine to benzocoumarin-3-carboxylic acid skeleton to provide efficient metal binding sites via the disposal of nitrogen and oxygen heteroatoms. Coumarin derivative was chosen as the fluorophore due to its excellent photochemical and photophysical properties, such as high fluorescence quantum yield and high stability against light [60]. The structure of **L** was confirmed by NMR and HRMS.

### 3.2. Spectroscopic Properties of L-Cu^2+^ Ensemble

The specific complexation of **L** with Cu^2+^ was firstly verified in detail through UV-vis absorption spectra. **L** exhibited a major absorption band centered at 378 nm in the DMF/HEPES mixed solution (7:3, *v/v*, pH = 7.4). Upon gradual addition of Cu^2+^ (0–60 μM) to the solution of **L**, the absorption peak at 378 nm steadily increased, indicating the formation of the L-Cu^2+^ ensemble (Figure S4A). To verify the specific coordination property of ligand **L** to Cu^2+^, equal amounts of other common metal ions including Pb^2+^, Ba^2+^, Ag^+^, Al^3+^, Cd^2+^, Ca^2+^, Mg^2+^, Co^2+^, Fe^3+^, Cr^2+^, Ni^2+^, Hg^2+^, Li^+^, Na^+^, K^+^, Zn^2+^, were added to the solution of **L**, respectively. It was exciting to observe that there were no obvious changes on absorption at 378 nm (Figure S4B), indicating the specific bond of **L** to Cu^2+^.

**Figure 1:**
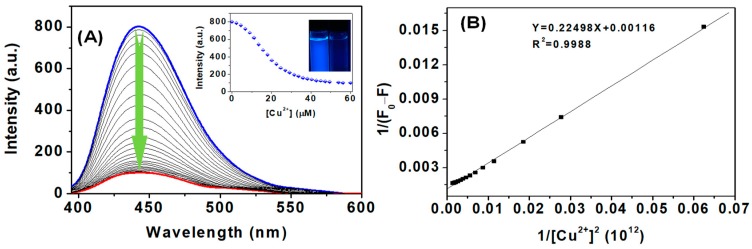
(**A**) Fluorescence spectra of **L** (10 μM) in the presence of different amounts of Cu^2+^ (0–60 μM) in the N,N-dimethylformamide (DMF)/HEPES mixed solution (7:3, *v/v*, Ph = 7.4). Insert: normalized fluorescence intensities of **L** at 443 nm as a function of Cu^2+^ concentration, and the changes of fluorescence colour in the absence and presence of Cu^2+^. (**B**) Benesi–Hildebrand linear analysis plot of **L** at different Cu^2+^ concentration. F_0_ and F represent the maximum emission of **L** at 443 nm in the absence and in the presence of Cu^2+^. Excitation was performed at 378 nm.

In the DMF/HEPES mixed solution (7:3, *v/v*, pH = 7.4), **L** exhibited strong blue fluorescence (Φ_1_ = 0.42) and could remain stable for at least 12 h (Figure S5), while the fluorescence intensity decreased gradually when certain amounts of paramagnetic Cu^2+^ were added. As shown in Figure 1A, the fluorescence intensity of **L** maximum at 443 nm quenched and reached a constant value accompanied by 88% fluorescence quenched (Φ_2_ = 0.052) when 60 μM Cu^2+^ was added. Job’s plots of the fluorescence emission variation at443 nm against the mole fraction of fluorescent ligand **L** clearly showed the inflection point at 0.67 (Figure S6), suggesting the formation of **L**-Cu^2+^ ensemble with a 2:1 binding stoichiometry [61]. It is speculated that one [62,63,64]. According to 2:1 binding mode, the association constant (*K*_a_) of **L** with Cu^2+^ was determined to be 7.28 × 10^9^ M^−2^ using a Benesi–Hildebrand plot (Figure 1B) [65].

**Figure 2:**
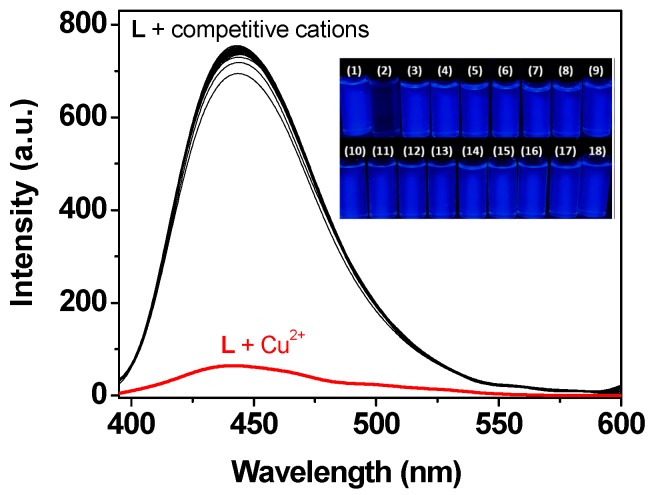
Fluorescence responses of **L** (10 μM) in the DMF/HEPES mixed solution (7:3, *v/v*, pH = 7.4) various cations (60 μM): (1) Free **L**, (2) Cu^2+^, (3) Pb^2+^, (4) Cd^2+^, (5) Co^2+^, (6) Ni^2+^, (7) Mn^2+^, (8) Hg^2+^, (9) Ag^+^, (10) Fe^3+^, (11) Cr^3+^, (12) Al^3+^, (13) Ba^2+^, (14) Ca^2+^, (15) Mg^2+^, (16) Li^+^, (17) Na^+^, (18) K^+^. Excitation at 378 nm.

The Cu^2+^-specific bond of **L** over various competitive metal ions was also verified under the same experimental conditions. As shown in Figure 2, nearly no emission changes were found after the addition of Pb^2+^, Ba^2+^, Ag^2+^, Al^3+^, Cd^2+^, Zn^2+^, Ca^2+^, Mg^2+^, Co^2+^, Mn^2+^, Fe^3+^, Cr^2+^, Ni^2+^, Hg^2+^, Li^+^, Na^+^, K^+^. The specific bond of **L** to Cu^2+^ was also confirmed by a “naked-eye” fluorescence colorimetric assay. It was found that the blue fluorescent colour quenched exclusively in the presence of Cu^2+^ (Figure 2, inset). The results indicated that **L** specifically binds to Cu^2+^ over other common metal ions.The effect of pH on the bonding ability between **L** and Cu^2+^ was then evaluated. Figure S7 showed that the fluorescence intensity of **L** was stable over a broad pH range from 4.5 to 11.5. However, after coordination with Cu^2+^, the fluorescence intensity decreased significantly to being very weak despite the negligible effect of acid (pH = 5.0) and alkali (pH = 11.0). It deserved to point out that the emission intensity of **L**-Cu^2+^ remained constant in the pH range of 5.0-11.0, which ensured the **L**-Cu^2+^ ensemble can be used as a potential fluorescence chemosensor for the detection of biothiols in the following experiments.

### 3.3. Spectroscopic Responses of L-Cu^2+^ towards Biothiols

**Figure 3:**
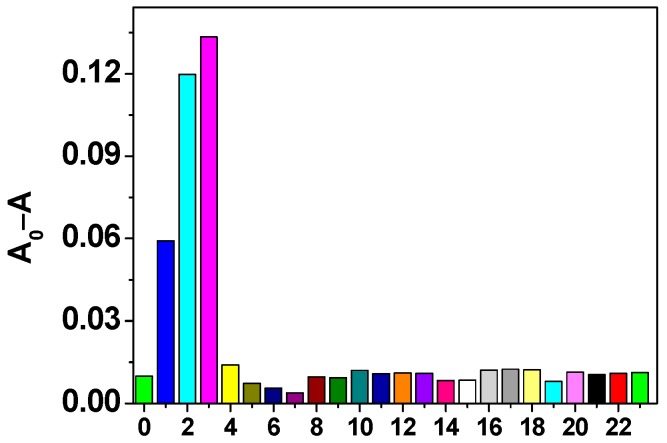
Ultraviolet-visible (UV-vis) absorption spectra of **L**-Cu^2+^ (10 μM) in the DMF/HEPES mixed solution (7:3, *v/v*, pH = 7.4) upon addition of various analytes (70 μM): 0. Blank, 1. Cys, 2. Hcy, 3. GSH, 4. acetylcysteine, 5. thiophenol, 6. sulfide, 7. bisulfate, 8. Val, 9. Ala, 10. Met, 11. Pro, 12. Thr, 13. Trp, 14. Gly, 15. Lys, 16. Phe, 17. Ser, 18. Asn, 19. His, 20. Gln, 21. Leu, 22. Try, 23. Arg. The absorbance values of **L**-Cu^2+^ were recorded at 378 nm.

On the basis of displacement strategy, the fluorescence sluggish **L**-Cu^2+^ ensemble, could be used as a platform for the real-time detection of biothiols by virtue of high affinity of sulfur towards copper [66,67]. The chemosensing ensemble, **L**-Cu^2+^ was prepared in situ by mixing **L** and Cu^2+^ in a 2:1 ratio in the DMF/HEPES mixed solution (7:3, *v/v*, pH = 7.4) solution. The absorption titration experiments of biothiols were firstly investigated. The changes in absorbance values (A_0_-A) of **L**–Cu^2+^ promoted by the addition of 70 μM of various amino acids and biothiols including valine (Val), alanine (Ala), methionine (Met), proline (Pro), threonine (Thr), tryptophan (Trp), glycine (Gly), lysine (Lys), phenylalanine (Phe), serine (Ser), asparagine (Asn), histidine (His), glutamine (Gln), leucine (Leu), tryptophan (Try), arginine (Arg), acetylcysteine, thiophenol, sulfide, bisulfate and glutathione (GSH), homocysteine (Hcy), cysteine (Cys), were evaluated. As shown in Figure 3, Cys, Hcy and GSH displayed remarkable responses in the absorption spectra. However, no obvious changes in UV–vis spectra were observed after the addition of other competitive species, demonstrating that **L**-Cu^2+^ has an excellent selectivity toward Cys, Hcy and GSH over other competitive small biological molecules. The sensing properties of **L**-Cu^2+^ towards biothiols were further studied by UV-vis titration experiments. As shown in Figure S8, upon the addition of Cys, Hcy and GSH to the solution of **L**-Cu^2+^ ensemble led to remarkable decrease of the absorption band centered at 378 nm. The final absorption spectra of the titration solutions were similar to free ligand **L** under the identical condition, indicating that the proposed displacement strategy for biothiols sensing was successfully achieved.

**Figure 4:**
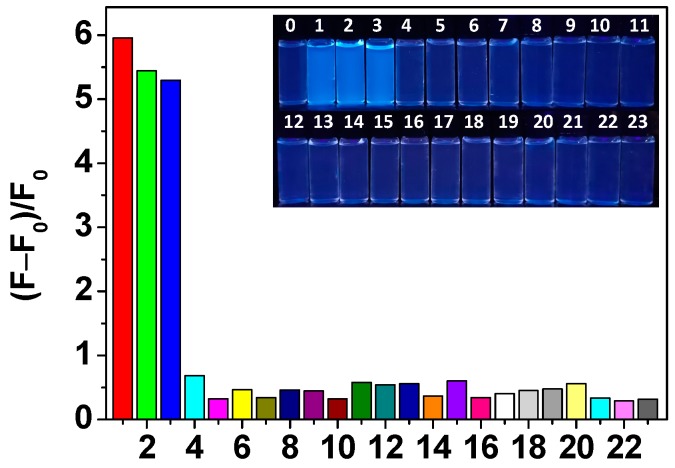
Normalized fluorescence responses of **L**-Cu^2+^ (10 μM) in the DMF/HEPES mixed solution (7:3, *v/v*, Ph = 7.4) in the presence of various analytes (70 µM): 1. GSH, 2. Hcy, 3. Cys, 4. acetylcysteine, 5. thiophenol, 6. sulfide, 7. bisulfate, 8. Val, 9. Ala, 10. Met, 11. Pro, 12. Thr, 13. Trp, 14. Gly, 15. Lys, 16. Phe, 17. Ser, 18. Asn, 19. His, 20. Gln, 21. Leu, 22. Try, 23. Arg. The intensities were recorded at 443 nm, excitation at 378 nm. Inset: the fluorescence colour responses of **L**-Cu^2+^ (10 μM) in the presence of various analytes (70 µM) under 365 nm UV light.

The selectivity of **L**-Cu^2+^ towards biothiols was further ensured by the measurement of fluorescence spectra. Figure 4 showed the ratio of fluorescence intensities enhancement ((F-F_0_)/F_0_) at 443 nm upon addition of various bioactive species. The addition of Cys, Hcy and GSH into **L**-Cu^2+^ solution in HEPES buffer led to 5.3-fold, 5.5-fold and 5.9-fold fluorescence enhancement, respectively. In contrast, other competitive species such as II-Leu, Ala, Arg, Asn, Asp, Gln, Glu, Gly, His, Leu, Lys, Met, Phe, Pro, Ser, Thr, Try, Val, and acetylcysteine, thiophenol, sulfide, bisulfate induced negligible fluorescence intensity changes.

**Figure 5:**
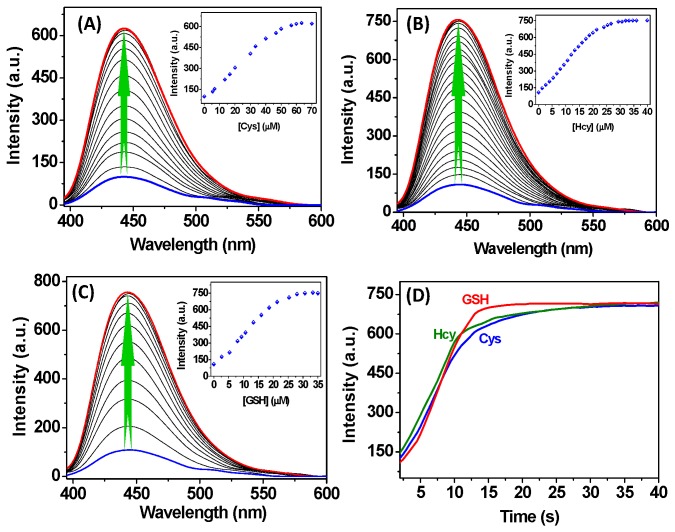
Fluorescence spectra of **L**-Cu^2+^ (10 μM) in the DMF/HEPES mixed solution (7:3, *v/v*, pH = 7.4) upon incremental addition of (**A**) Cys (0-70 μM), (**B**) Hcy (0-40 μM) and (**C**) GSH (0-35 μM). Inset: the plots of the fluorescence intensity of **L**-Cu^2+^ at 443 nm as the function of biothiols concentrations, respectively. (**D**) Time-dependent fluorescence intensity of **L**-Cu^2+^ (10 μM) at 443 nm upon incremental addition of Cys (0-70 μM), Hcy (0-40 μM) and GSH (0-35 μM) in the DMF/HEPES mixed solution (7:3, *v/v*, pH = 7.4). The intensities were recorded at 443 nm, excitation was performed at 378 nm.

Furthermore, fluorescence titration analysis of Cys, Hcy and GSH with **L**-Cu^2+^ ensemble was also studied. It can be clearly shown in Figure 5A–C that **L**-Cu^2+^ maintained an emission “OFF” state in the DMF/HEPES mixed solution (7:3, *v/v*, pH = 7.4). With increasing amounts of biothiols, the emission of **L**-Cu^2+^ maximum at 443 nm progressively intensified when 70 μM Cys, 40 μM Hcy and 35 μM GSH were added.The fluorescence spectra of **L**-Cu^2+^ with biothiols were similar to that of the free ligand **L**, which could prove the sensing mechanism that ligand **L** was released from the chemosensing ensemble (**L**-Cu^2+^) owing to the strong affinity of biothiols with Cu^2+^. And, the fluorescence quantum yields of **L**-Cu^2+^ upon the addition of Cys, Hcy and GSH were calculated to be Φ_3_ = 0.33, Φ_4_ = 0.39 and Φ_5_ = 0.40, respectively. Additionally, a good linearity was found between fluorescence intensity of **L**-Cu^2+^ at 443 nm versus the concentrations of Cys, Hcy and GSH (Figure S9), and the detection limits were determined to be 0.96 μM, 0.68 μM and 0.44 μM based on 3*σ*/slope according to reported method [68,69]. Fluorescence responses times of **L**-Cu^2+^ towards biothiols were also discussed in Figure 5D. It took around 15 s for GSH to displace Cu^2+^, while this process lasted for around 20 s when it came to Cys and Hcy. In a brief summary, the results indicated that **L**-Cu^2+^ can be used for real-time detection of biothiols.

**Figure 6:**
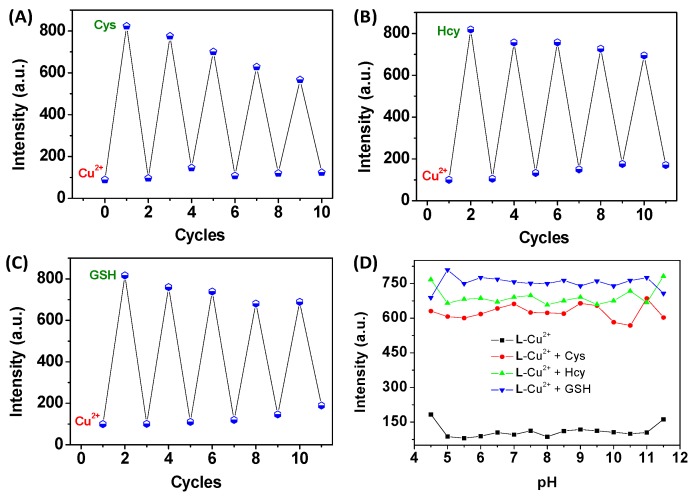
Fluorescence changes of **L**-Cu^2+^ (10 μM) at 443 nm in the DMF/HEPES mixed solution (7:3, *v/v*, pH = 7.4) upon the alternate addition of (**A**) Cu^2+^/Cys, (**B**) Cu^2+^/Hcy and (**C**) Cu^2+^/GSH with several concentration ratios. Cu^2+^/Cys: 0:0, 10:0, 10:10, 20:10, 20:20, 30:20; 30:30, 40:30, 40:40, 50:40. Cu^2+^/Hcy: 0:0, 10:0, 10:10, 20:10, 20:20, 30:20; 30:30, 40:30, 40:40, 50:40. Cu^2+^/GSH: 0:0, 10:0, 10:5, 20:5, 20:10, 30:10; 30:20, 40:20, 40:30, 50:30. (**D**) Influence of pH on the fluorescence intensities of **L**-Cu^2+^ in the DMF/HEPES mixed solution (7:3, *v/v*, pH = 7.4) in the absence and presence of biothiols. The intensities were recorded at 443 nm, excitation was performed at 378 nm.

Since regeneration is a key factor to evaluate ensemble based chemosensor, the reversible “OFF-ON-OFF” fluorescence responses of **L**-Cu^2+^ were conducted by alternative addition of biothiols and Cu^2+^ in the DMF/HEPES mixed solution (7:3, *v/v*, pH = 7.4). As can be seen in (Figure 6A–C), switchable changes in the emission intensity at 443 nm could be repeated 5 times at least, implying **L**-Cu^2+^ was a reversible chemosensor for the detection of biothiols under physiological conditions. Figure 6D discussed the effect of pH on sensing performance of **L**-Cu^2+^ towards biothiols. It was clearly found that the fluorescence intensities of **L**-Cu^2+^ at 443 nm kept constant values at a pH range of 5.0–11.0, indicating its reliability under the test condition. The addition of biothiols in **L**-Cu^2+^aqueous solution, as expected, led to remarkable fluorescence enhancement from pH 5.0 to pH 11.0. The result indicated that **L**-Cu^2+^ can be used for the detection of biothiols in a broad pH range.

### 3.4. ‘Naked-Eye’ Detection of Biothiols in Human Urine Samples

**Figure 7:**
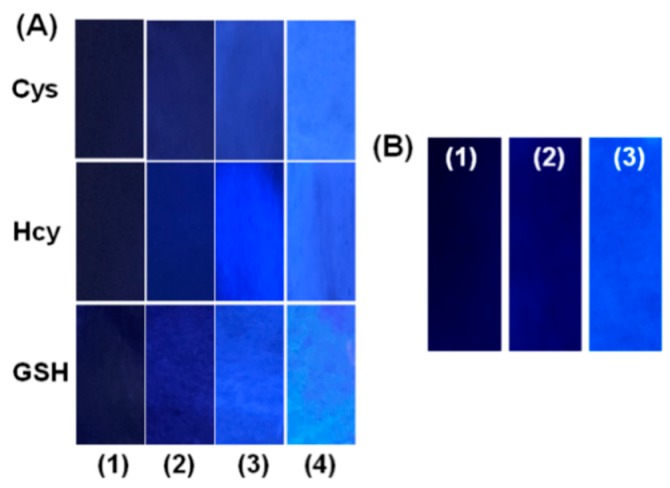
(**A**) Photographs of fluorescence colour responses of the **L**-Cu^2+^-coated test papers immersed to different concentration of Cys, Hcy and GSH in water under 365 nm UV light: (1) 0 mM, (2) 0.05 mM, (3) 0.5 mM, (4) 5 mM. (**B**) Photographs of the fluorescence colour responses in human urine sample: (1) **L**-Cu^2+^-coated test paper only, (2) Free filter paper exposed to pure urine, (3) **L**-Cu^2+^-coated test paper when immersed to human urine under 365 nm UV light. All the filter paper was exposed to the samples for 30 seconds.

In regards to the excellent properties of **L**-Cu^2+^ for the detection of biothiols (Table S1), the practical application of **L**-Cu^2+^ for the “naked-eye” detection of biothiols in human urine sample levels was then verified by using **L**-Cu^2+^-coated filter paper. The tests were prepared by putting filter papers into the acetonitrile solution of **L**, followed by dipping into Cu^2+^ solution. Then, **L**-Cu^2+^-coated test papers were dried using the blower. We firstly studied the feasibility of the test paper for the “naked-eye” detection of biothiols in pure water. The papers were immersed in the test aqueous solution containing different concentrations of Cys, Hcy and GSH. As shown in Figure 7A, strong blue fluorescence emerged and became brighter with increasing biothiols concentrations under 365 nm UV light. The results indicated that the **L**-Cu^2+^-coated test paper has the potential to be used for the “naked-eye” detection of biothiols levels in practical body fluid samples. Clinically, patients are always supposed to do uroscopy for diagnosing diseases and biothiols content was one of key indexes due to their easily oxidizable nature. Therefore, we hypothesized about the possibility of **L**-Cu^2+^-coated test paper on the detection of biothiols in human urines. To eliminate the possible impact of urine self-fluorescence, urine immersed filter paper was photographed first under 365 UV light. Although weak fluorescence emitted from urine-immersed **L**-Cu^2+^-blank test paper, it still can be neglected when compared to those **L**-Cu^2+^-coated test paper immersed in normal urine (Figure 7B). Consequently, **L**-Cu^2+^-coated test paper has the potential for “naked-eye” detection of biothiols in real body fluids.

## 4. Conclusions

In summary, a novel fluorescence chemosensor (**L**-Cu^2+^) based on a benzocoumarin-Cu^2+^ ensemble has been developed for the detection of biothiols (Cys, Hcy and GSH). The 2:1 stoichiometry structure of **L**-Cu^2+^ was demonstrated by Job’s plot and the Benesi–Hildebrand plot. **L**-Cu^2+^ was successfully used as an “OFF-ON” chemosensor for the detection of Cys, Hcy and GSH based on the displacement approach, giving a remarkable recovery of fluorescence and UV-Vis spectra. The presented **L**-Cu^2+^ ensemble exhibited the advantages of simple synthesis, excellent stability under broad pH, and high selectivity. The fluorescence “OFF–ON” circles can be repeated more than 5 times by the alternative addition of biothiols and Cu^2+^, implying that **L**-Cu^2+^ is a renewable chemosensor. The practical application of **L**-Cu^2+^ for the “naked-eye” detection of biothiol levels in human urine sample was verified by using **L**-Cu^2+^-coated test paper. The successful investigations of **L**-Cu^2+^ ensemble in a practical body fluids indicated that the application has potential in the rapid disease diagnosis and monitoring treatment fields in the future.

## Supplementary Materials

The following are available online at https://www.mdpi.com/1424-8220/20/5/1331/s1: Scheme S1: Synthetic procedure of **L**, Figure S1: High-resolution mass spectrometry (HRMS) of **L**, Figures S2 and S3: NMR of **L**, Figure S4: UV-vis absorption spectra of **L** in the presence of cations, Figure S5: The stability of **L**, Figure S6: Job’s plot, Figure S7: Effect of pH on the fluorescence intensities of **L**, Figure S8: Absorption spectra of **L**-Cu^2+^ in the presence of different amounts of biothiols, Figure S9: Linear relationship of **L**-Cu^2+^ versus the concentration of biothiols, Table S1: Comparison of this work with reported fluorescent chemosensors for biothiols detection.
