# Supplementary material for: A Copper (II) Ensemble-Based Fluorescence Chemosensor and Its Application in the ‘Naked–Eye’ Detection of Biothiols in Human Urine
Source: Sensors (Basel). 2020 Feb 29;20(5):1331. doi: 10.3390/s20051331 (PMC7085593; doi:10.3390/s20051331)
Supplement: Supplementary file 1 [file sensors-20-01331-s001.pdf]

Supplementary Materials

A Copper (II) Ensemble Based Fluorescence Chemosensor and the Application in the "Naked–eye" Detection of Biothiols in Human Urine

Yue Wang[1], Huan Feng[1], Haibo Li[2], Xinyi Yang[1], Hongmin Jia[1], Wenjun Kang[2], Qingtao Meng[1,]*, Zhiqiang Zhang[1,]* and Run Zhang[3]

1. School of Chemical Engineering, University of Science and Technology Liaoning, Anshan, Liaoning, 114051, P. R. China; Wangyue9088@163.com (Y.W.); 18369956613@163.com (H.F.); yxz0601y@163.com (Y.Y.); jhongmin66@163.com (H.J.)
2. Shandong Provincial Key Laboratory of Chemical Energy Storage and Novel Cell Technology, Department of Chemistry, Liaocheng University, Liaocheng, 252059, China; haiboli@mail.ustc.edu.cn (H.L.); kangwenjun@lcu.edu.cn (W.K.)
3. Australian Institute for Bioengineering and Nanotechnology, The University of Queensland, Brisbane, 4072, Australia; r.zhang@uq.edu.au (R.Z.)

* Correspondence: qtmeng@ustl.edu.cn (Q.M.); zhangzhiqiang@ustl.edu.cn (Z.Z.); Tel.: +86-412-5929627 (Q.M.)

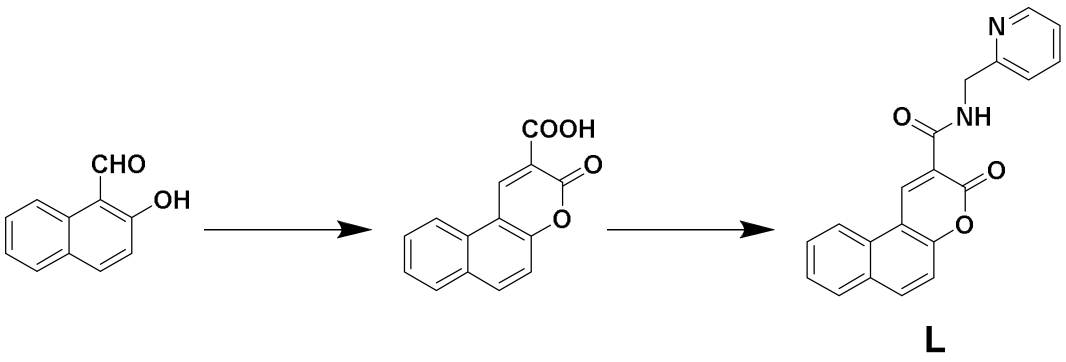

Scheme S1. Synthetic procedure of the fluorescent ligand, **L**

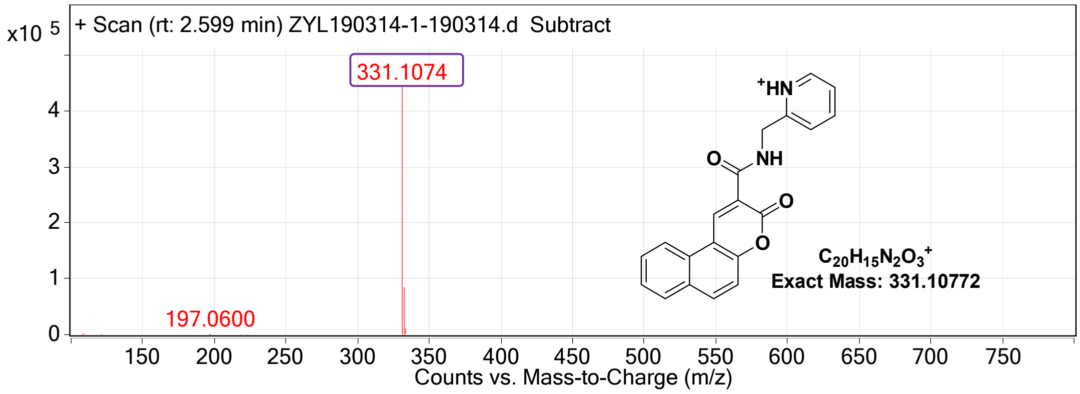

Figure S1. HRMS of fluorescent ligand, **L**.

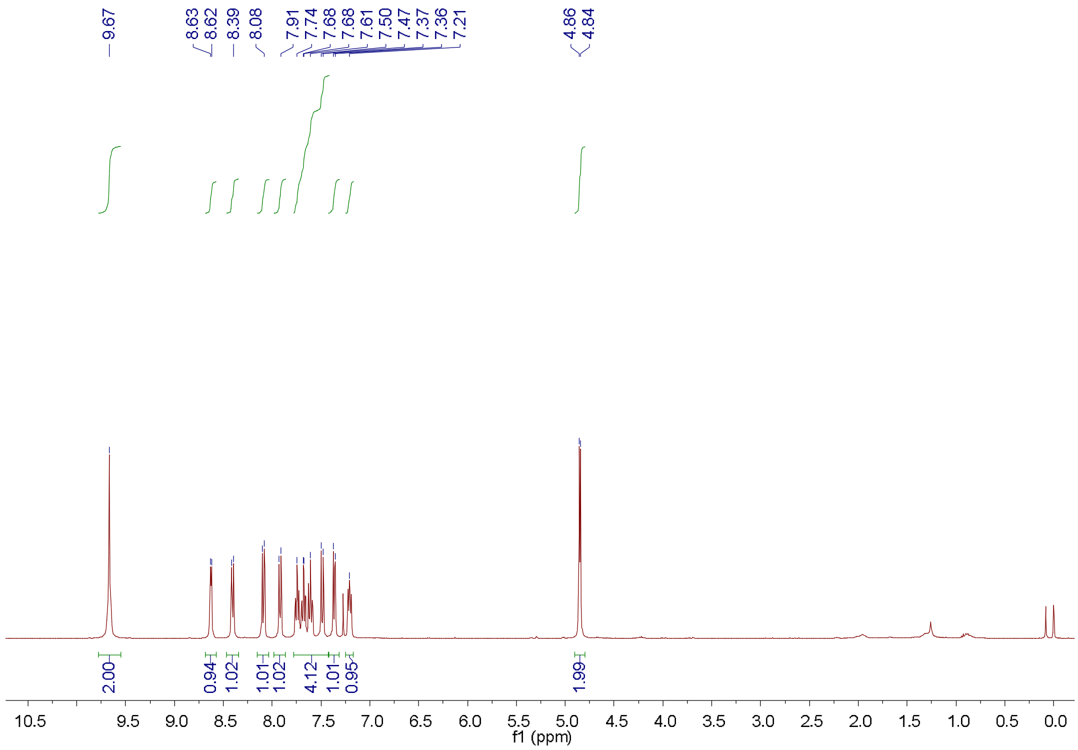

Figure S2. ^{1}H NMR of fluorescent ligand, **L** ($CDCl_3$, 600 MHz)

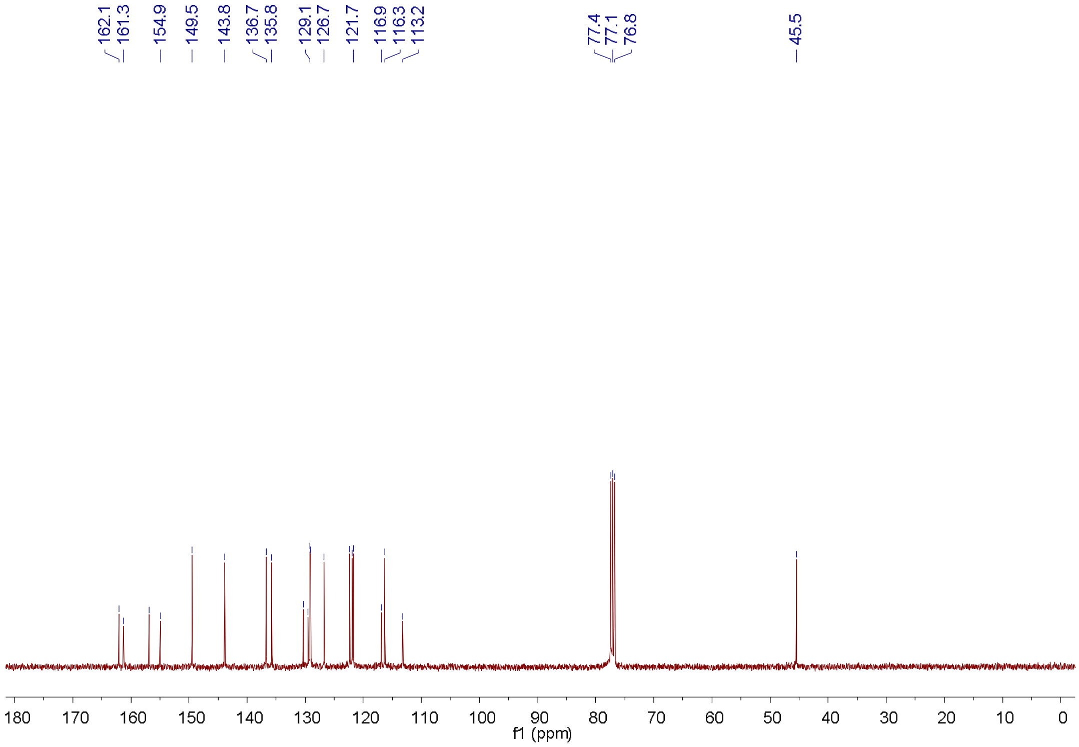

Figure S3. ^{13}C NMR of fluorescent ligand, **L** ($CDCl_3$, 150 Hz).

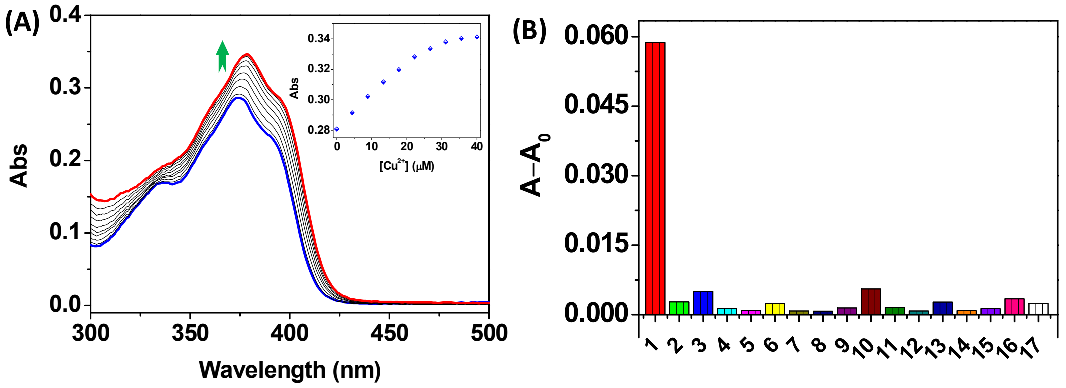

Figure S4. (A) UV-vis absorption spectra of **L** (10 μM) in the presence of different amounts of Cu^{2+} (0–60 μM) in the DMF/HEPES mixed solution (7:3, v/v, pH=7.4). Inset: normalized absorption intensities of **L** at 378 nm as a function of Cu^{2+} (0–60 μM). (B) UV-vis absorption spectra of **L** (10 μM) in the DMF/HEPES mixed solution (7:3, v/v, pH=7.4) upon addition of 60 μM various cations: 1. Cu^{2+}, 2. Pb^{2+}, 3. Ba^{2+}, 4. Ag^{2+}, 5. Al^{3+}, 6. Cd^{2+}, 7. Ca^{2+}, 8. Mg^{2+}, 9. Co^{2+}, 10. Fe^{3+}, 11. Cr^{2+}, 12. Ni^{2+}, 13. Hg^{2+}, 14. Li^{+}, 15. Na^{+}, 16. K^{+}, 17. Zn^{2+}. A_0 and A represent the absorption intensities of **L** at 378 nm in the absence and in the presence of Cu^{2+}.

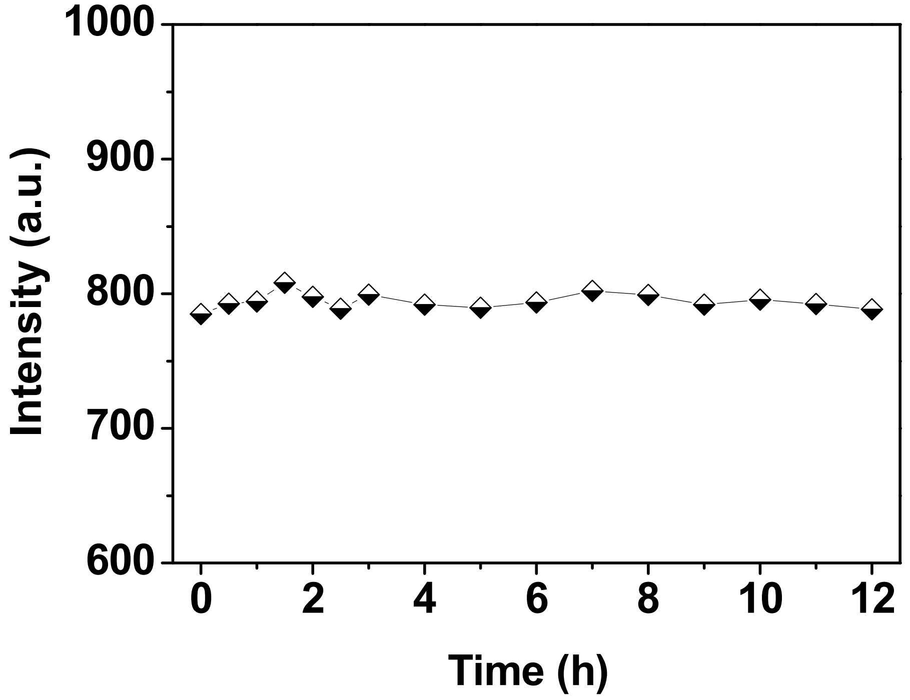

Figure S5. Fluorescence of **L** (10 μM) at different times in the DMF/HEPES mixed solution (7:3, v/v, pH=7.4). The intensities were recorded at 443 nm, and excitation was performed at 378 nm.

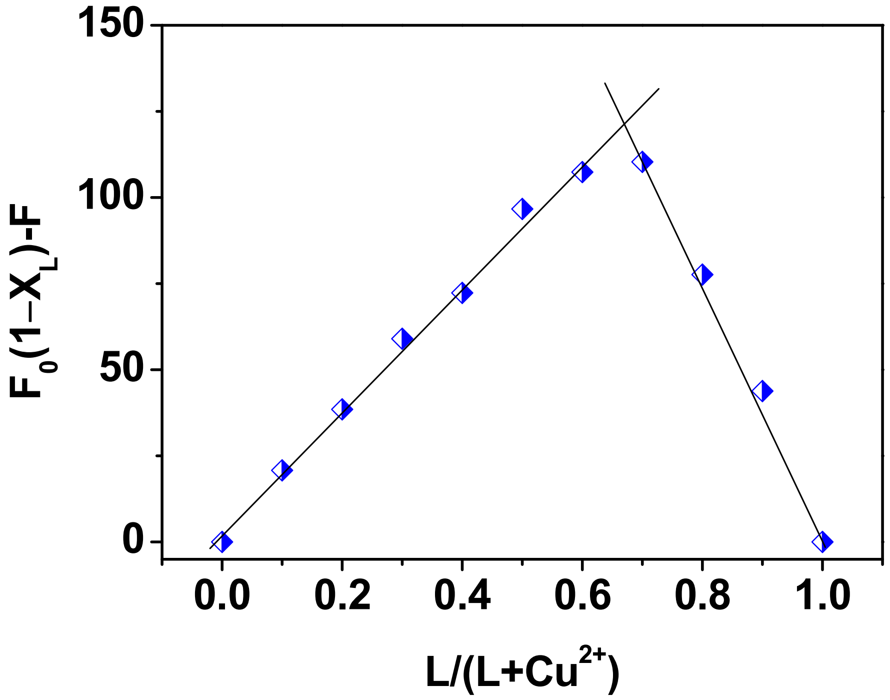

Figure S6. Job's plots according to the method for continuous variations. The total concentration of **L** (10 μM) and Cu^{2+} is 10 μM. X_L represents the proportion of **L** to the total amounts of **L** and Cu^{2+}. The intensities were recorded at 443 nm, and excitation was performed at 378 nm.

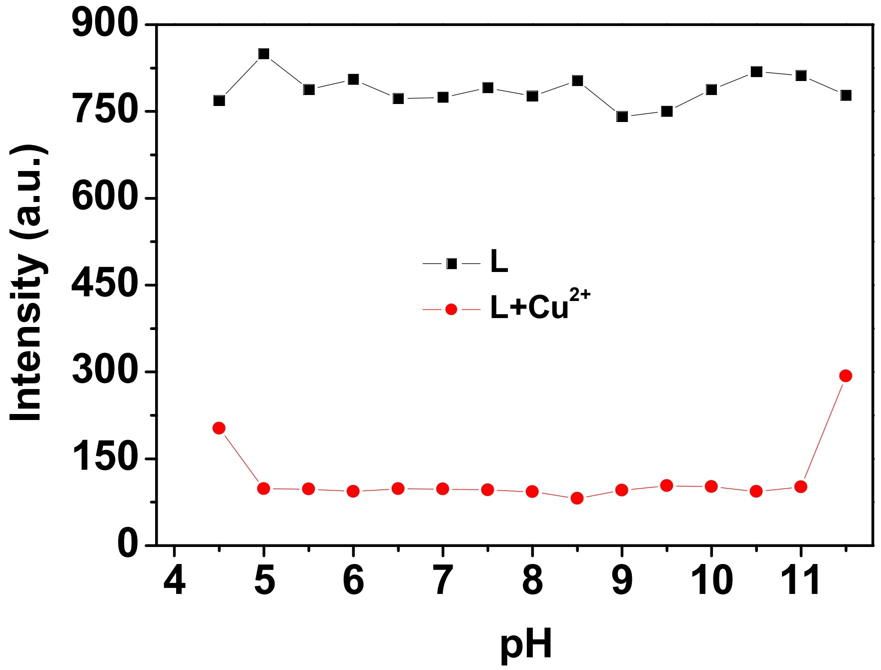

Figure S7. Effect of pH on the fluorescence intensities of **L** (10 μM) in the absence and presence of Cu^{2+} (60 μM) in the DMF/H_2O mixed solution. The intensities were recorded at 443 nm, and excitation was performed at 378 nm.

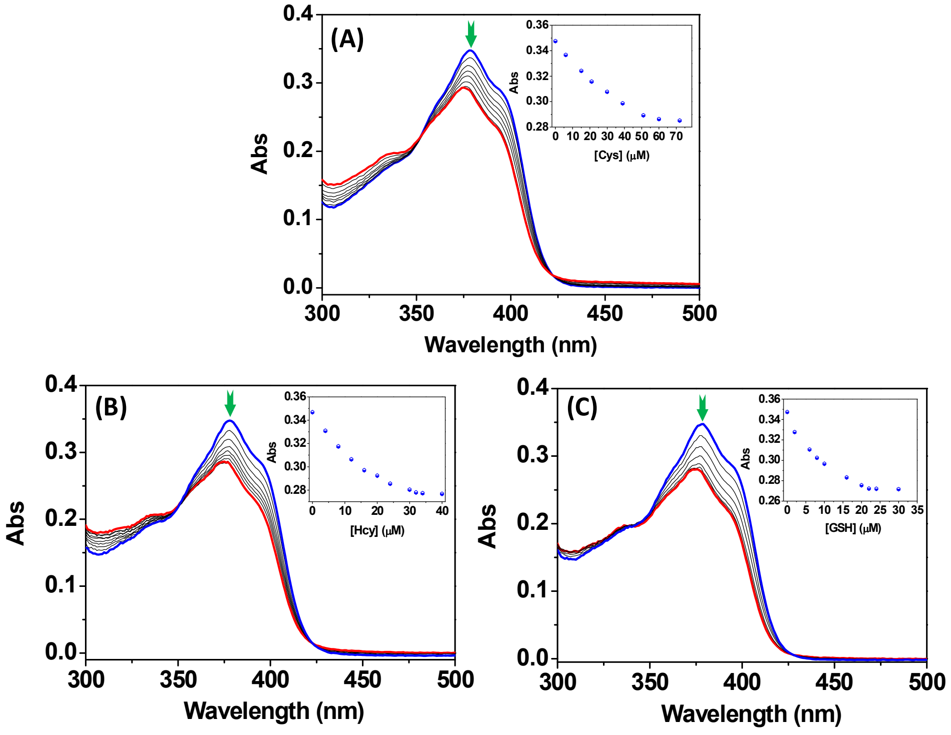

Figure S8. Absorption spectra of **L**-Cu^{2+} (10 μM) in the presence of different amounts of Cys (0–70 μM), Hcy (0–40 μM), GSH (0–30 μM) in the DMF/HEPES mixed solution (7:3, v/v, pH=7.4).

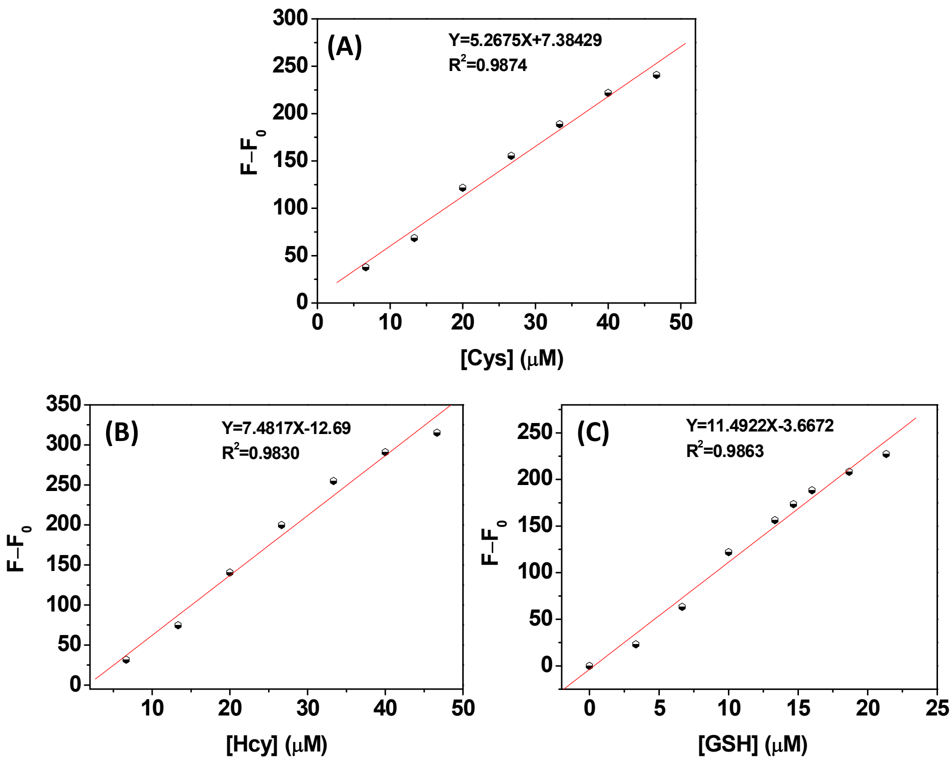

Figure S9. The linear relationship between fluorescence intensity of **L**-Cu^{2+}(1 μM) at 443 nm versus the concentration of (A) Cys, (B) Hcy and (C) GSH in the DMF/HEPES mixed solution (7:3, v/v, pH=7.4). Excitation was performed at 378 nm. Intensities were recorded at 443 nm.

Table S1. Comparison of this work with reported fluorescent chemosensors for biothiols detection.

Probes	Selectivity	Detection limit	Response time	Detection of Biothiols by "Naked–eye"	Detection of biothiols in Human Urine	Test papers	Ref.
**RhAN**	GSH	0.1 μM	< 5 s	Yes	No	No	S1
**probe**	biothiols	GSH 3.7 μM	~ 120 min	No	No	No	S2
**Compoud 1**	biothiols	GSH 10.3 nM	< 5 s	Yes	No	No	S3
**Probe1**	Cys	1.4 nM	~ 30 min	No	No	No	S4
**SATZ**	Cys/Hcy	2.843 μM (Cys)	12 min	Yes	No	No	S5
**CI**	Cys<br>Hcy<br>GSH	0.014 μM<br>0.081 μM<br>0.097 μM	25 min<br>55 min<br>50 min	Yes	No	No	S6
**BT-AC**	Cys	36.2 nM	--	No	No	test strip	S7
**CPR**	Cys<br>Hcy<br>GSH	12 nM<br>13 nM<br>30 nM	40 min<br>100 min<br>38 min	No	No	No	S8

**NIRHA**	Cys	77.6 μM	15 min	Yes	No	No	S9
**SHCy-C**	Cys	21.2 nM	5 min	No	No	No	S10
**HN-NBD**	Cys<br>Hcy<br>GSH	31 μM<br>66 μM<br>58 μM	5 min	No	No	No	S11
**CPA**	Cys<br>Hcy	49 nM<br>51 nM	~3 min	No	No	No	S12
**Nap-Cys**	Cys	1.8 μM	5 min	No	No	No	S13
**PYR**	Cys<br>Hcy	22 nM<br>23nM	5min<br>10min	Yes	No	No	S14
**Lyso-RC**	Cys<br>Hcy<br>GSH<br>H_2S	27 nM<br>33 nM<br>16 nM	--	No	No	No	S15
**probe 1**	Cys<br>Hcy<br>GSH	Cys 0.11 μM	20 min	No	No	No	S16
**EuTc-H_2O_2**	Cys<br>Hcy<br>GSH	100 nM<br>200 nM<br>400 nM	--	No	Yes	No	S17

References:

1. Tong, L.; Qian, Y. A NIR rhodamine fluorescent chemodosimeter specific for glutathione: Knoevenagel condensation, detection of intracellular glutathione and living cell imaging. *J. Mater. Chem. B* **2018**, *6*, 1791-1798.

2. Gao, B.; Cui, L.; Pan, Y.; Zhang, G.; Zhou, Y.; Zhang, C.; Shuanga, S.; Dong, C. A highly selective ratiometric fluorescent probe for biothiol and imaging in live cells. *RSC Adv.* **2016**, *6*, 43028-43033.

3. Zhang, M.; Han, H.; Zhang, S.; Wang, C.; Lu, Y.; Zhu, W. A new colorimetric and fluorescent probe with a large stokes shift for rapid and specific detection of biothiols and its application in living cells. *J. Mater. Chem. B* **2017**, *5*, 8780-8785.

4. Chen, X.; Xu, H.; Ma, S.; Tong, H.; Lou, K.; Wang, W. A simple two-photon turn-on fluorescent probe for the selective detection of cysteine based on a dual PeT/ICT mechanism. *RSC Adv.* **2018**, *8*, 13388-13392.

5. Song, H.; Zhou, Y.; Qu, H.; Xu, C.; Wang, X.; Liu, X.; Zhang, Q.; Peng, X. A Novel AIE Plus ESIPT Fluorescent Probe with a Large Stokes Shift for Cysteine and Homocysteine: Application in Cell Imaging and Portable Kit. *Ind. Eng. Chem. Res.* **2018**, *57*, 15216−15223.

6. Xu, G.; Tang, G.; Lin, W. A multi-signal fluorescent probe for the discrimination of

cysteine/homocysteine and glutathione and application in living cells and zebrafish. *New J. Chem.* **2018**, *42*, 12615-12620

7. Zhu, M.; Wu, X.; Sang, L.; Fan, F.; Wang, L.; Wu, X.; Hua, R.; Wang, Y.; Qing X. Li. A novel and effective benzo[d]thiazole-based fluorescent probe with dual recognition factors for highly sensitive and selective imaging of cysteine in vitro and in vivo. *New J. Chem.* **2019**, *43*, 13463-13470.

8. Zhang, H.; Wang, B.; Ye, Y.; Chen, W.; Song, X. A ratiometric fluorescent probe for simultaneous detection of Cys/Hcy and GSH. *Org. Biomol. Chem.* **2019**, *17*, 9631-9635.

9. Qi, S.; Zhu, L.; Wang, X.; Du, J.; Yang, Q.; Li, Y. Near-infrared turn-on fluorescent probe for discriminative detection of Cys and application in in vivo imaging. *RSC Adv.* **2019**, *9,* 41431-41437.

10. Cai, S.; Liu, C.; Jiao, X.; Zhao, L.; Zeng, X. Lysosome-targeted Near-Infrared Fluorescence Probe for Imaging Endogenous Cysteine (Cys) in Living Cells. *J. Mater. Chem. B* DOI: 10.1039/C9TB02609F

11. Zhu, H.; Zhang, H.; Liang, C.; Liu, C.; Jia, P.; Li, Z.; Yu, Y.; Zhang, X.; Zhu, B.; Sheng,W. A novel highly sensitive fluorescent probe for bioimaging biothiols and its applications in distinguishing cancer cells from normal cells. *Analyst* **2019**, *144*, 7010-7016.

12. Cheng, T.; Huang, W.; Gao, D.; Yang, Z.; Zhang, C.; Zhang, H.; Zhang, J.; Li, H.; Yang, X. Michael Addition/S,N-Intramolecular Rearrangement Sequence Enables Selective Fluorescence Detection of Cysteine and Homocysteine. *Anal. Chem.* **2019**, *91*, 10894−10900.

13. Dong, B.; Lu, Y.; Zhang, N.; Song, W.; Lin, W. Ratiometric Imaging of Cysteine Level Changes in Endoplasmic Reticulum during H2O2‑Induced Redox Imbalance. *Anal. Chem.* **2019**, *91*, 5513−5516.

14. Yang, M.; Fan, J.; Sun, W.; Du, J.; Peng, X. Mitochondria-Anchored Colorimetric and Ratiometric Fluorescent Chemosensor for Visualizing Cysteine/Homocysteine in Living Cells and Daphnia magna Model. *Anal. Chem.* **2019**, *91*, 12531−12537.

15. Zhang, H.; Xu, L.; Chen, W.; Huang, J.; Huang, C.; Sheng, J.; Song, X. A Lysosome-Targetable Fluorescent Probe for Simultaneously Sensing Cys/Hcy, GSH, and H2S from Different Signal Patterns. *ACS Sens.* **2018**, *3*, 2513−2517

16. Nomura, N.; Nishihara, R.; Nakajima, T.; Kim, S.; Iwasawa, N.; Hiruta, Y.; Nishiyama, S.; Sato, M.; Citterio, D.; Suzuki, K. Biothiol-Activatable Bioluminescent Coelenterazine Derivative for Molecular Imaging in Vitro and in Vivo. *Anal. Chem.* **2019**, *91*, 9546−9553.

17. Xie, F.; Tan, H.; Li, Z.; Yang, H. A europium-based fluorescence probe for detection of thiols in urine. *Anal. Methods*, **2014**, *6*, 6990-6996
